# Cardiopulmonary Resuscitation With Mechanical Chest Compression Device During Percutaneous Coronary Intervention. A Case Report

**DOI:** 10.3389/fcvm.2021.614493

**Published:** 2021-06-10

**Authors:** Dóra Ujvárosy, Veronika Sebestyén, Tamás Ötvös, Balázs Ratku, István Lorincz, Tibor Szuk, Zoltán Csanádi, Ervin Berényi, Zoltán Szabó

**Affiliations:** ^1^Department of Emergency Medicine, Faculty of Medicine, University of Debrecen, Debrecen, Hungary; ^2^Doctoral School of Health Sciences, Faculty of Public Health, University of Debrecen, Debrecen, Hungary; ^3^Department of Cardiology, Faculty of Medicine, University of Debrecen, Debrecen, Hungary; ^4^Department of Radiology, Faculty of Medicine, University of Debrecen, Debrecen, Hungary

**Keywords:** sudden cardiac death, cardiopulmonary resuscitation, coronary intervention, mechanical chest compression device, case report

## Abstract

Sudden cardiac death is a leading cause of death worldwide, whereby myocardial infarction is considered the most frequent underlying condition. Percutaneous coronary intervention (PCI) is an important component of post-resuscitation care, while uninterrupted high-quality chest compressions are key determinants in cardiopulmonary resuscitation (CPR). In our paper, we evaluate a case of a female patient who suffered aborted cardiac arrest due to myocardial infarction. The ambulance crew providing prehospital care for sudden cardiac arrest used a mechanical chest compression device during advanced CPR, which enabled them to deliver ongoing resuscitation during transfer to the PCI laboratory located 20 km away from the scene. Mechanical chest compressions were continued during the primary coronary intervention. The resuscitation, carried out for 2 h and 35 min, and the coronary intervention were successful, as evidenced by the return of spontaneous circulation and by the fact that, after a short rehabilitation, the patient was discharged home with a favorable neurological outcome. Our case can serve as an example for the effective and safe use of a mechanical compression device during primary coronary intervention.

## Introduction

Sudden cardiac death is one of the leading causes of death (1), accounting for 20% of total mortality. In Europe, ~275,000 patients are affected annually (2), and their survival rate is <10% (3). Frequent underlying causes are myocardial infarction and pulmonary embolism (4, 5). The key to improving survival lies in the properly performed cardiopulmonary resuscitation (CPR) characterized by continuous, high-quality chest compressions and minimized interruptions (6–9). Under certain circumstances (e.g., transporting critically ill patients, treating hypothermic patients, in urgent need for coronary intervention), the use of mechanical compression devices can be beneficial (10–15).

The most widely used mechanical chest compression devices are the AutoPulse (AutoPulse Resuscitation System Model 100, Zoll, CA) and the LUCAS (Lund University Cardiopulmonary Assist System, JOLIFE AB Inc., Lund, Sweden) (16). Although these devices are different regarding their working principle, both of them are capable of delivering high-quality chest compressions (17, 18). Both offer chest compressions with appropriate rate and depth range, facilitate full recoil, prevent worsening CPR quality due to provider's fatigue, and ensure safe defibrillation (16). Chest recoil is an important and well-established condition for both successful resuscitation and the long-term neurological outcome (19–22). Comparative studies of the two devices have not found significant differences in the return of spontaneous circulation (ROSC) and in the nature or number of injuries they caused (16, 23, 24). Considering that myocardial infarction is the most frequent cause of sudden cardiac death, primary coronary intervention (PCI) became a core element of post-resuscitation care (25). Given the conflicting opinions on compression devices, we present our results through the story of our female patient who suffered sudden cardiac death due to myocardial ischemia, received mechanical chest compressions as a part of her emergency treatment, and the continuous mechanical circulatory support was maintained up to the completion of coronary intervention. Case reports from the National Ambulance Service of Hungary and medical documents recorded in the electronic system of the University of Debrecen were analyzed retrospectively.

## Case Presentation

In 2013, ambulance was dispatched to a 44-year-old woman. According to the call the patient had no spontaneous breathing. Based on the family's report, the patient had no known previous diseases apart from hypertension, which was treated with medications.

**Figure 1:**
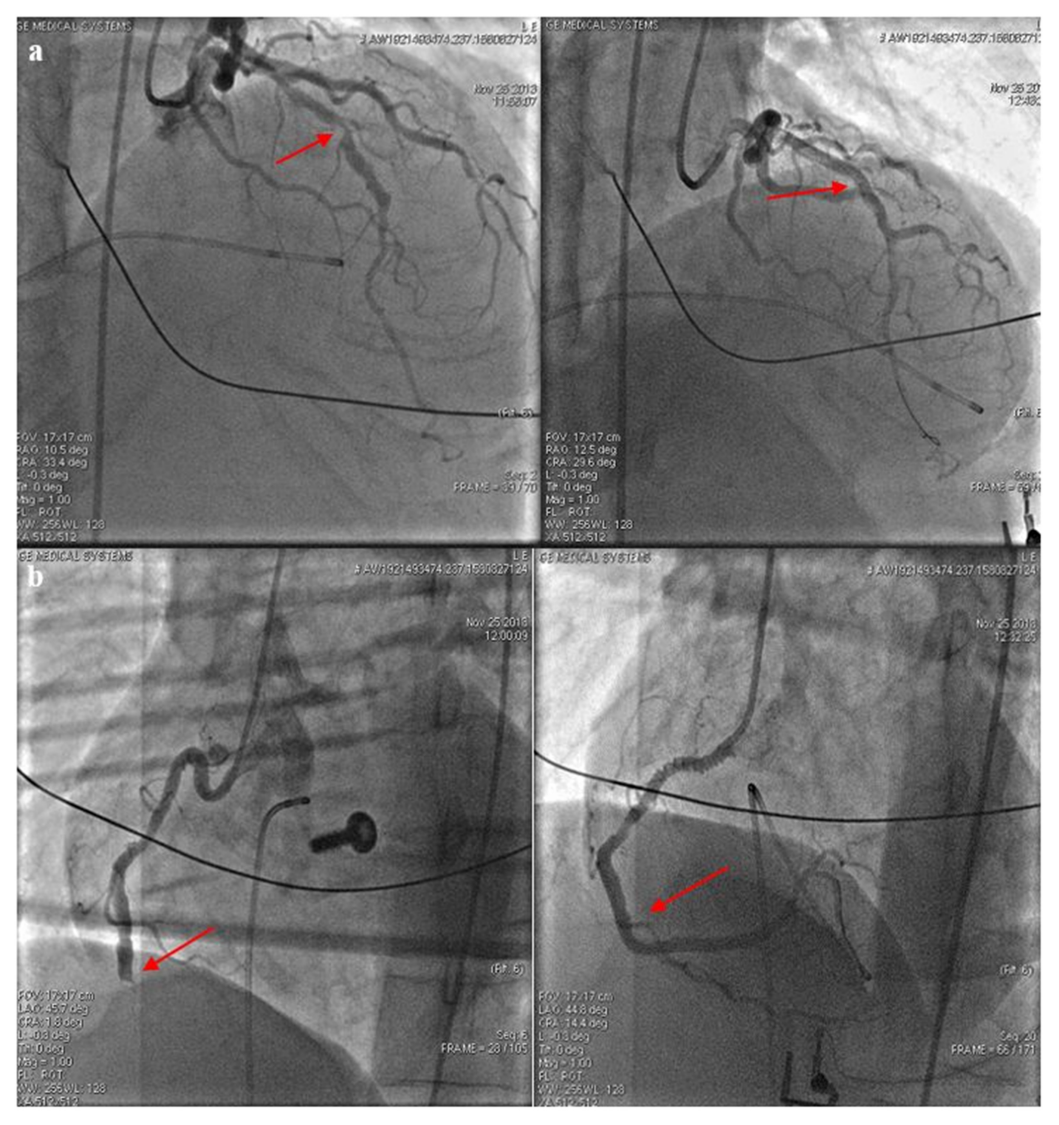
**(a)** The left anterior descending artery (LAD) before and after stent implantation. After predilation at 14 atm, a 3.5 × 30 mm Integrity stent was positioned into the stenosis of the LAD. Red arrows indicate the sites of intervention. Images taken before (left picture) and after (right picture) the intervention. **(b)** The right coronary artery (RCA) before and after stent implantation. During the intervention, a dissection developed at the extremely tortuous ostium of the RCA; to this site a 3.5 × 24 mm Omega stent was placed after predilation at 15 atm (marked with right arrow). A 3.5 × 12 mm Omega stent was implanted to the distal area resulting in a Thrombolysis In Myocardial Infarction (TIMI) grade 3 flow.

On the day of the call, at 8:45, she complained of chest pain and numbness in her left arm and then at 9:48, suddenly she lost her consciousness. The relatives called the emergency at 9:50 and the ambulance unit arrived at the scene 5 min later. Bystander CPR was not attempted; her family members did not even check her vital signs. During the primary assessment, the patient showed no signs of life, her pupils were dilated and non-reactive to light. At 9:56 the ambulance crew immediately started performing manual chest compressions. The initial rhythm proved to be ventricular fibrillation (VF); therefore, at 9:58 they performed defibrillation using an energy level of 200 J and continued the resuscitation by strictly adhering to the Advanced Life Support (ALS) cardiac arrest algorithm. LUCAS-2 mechanical chest compression device was applied and used in a continuous mode. Despite a total of five shocks (200–360–360 J), the VF still persisted. However, following the fifth shock, her rhythm changed to P-wave asystole. A total of 8 mg of epinephrine and 450 mg (300 + 150 mg) of amiodarone were given during the CPR. Considering the patient's initial complaints, acute coronary syndrome was suspected as the underlying condition. When the ambulance left the scene, mechanical chest compressions had already been carried out for 50 min. During the transport to the hospital, she received 500 ml of crystalloid solution and 2 g of magnesium sulfate; furthermore, 250 mg of dobutamine (3.6 μg/kg/h) and 100 mg of dopamine (8.2 μg/kg/h) were administered in a continuous infusion due to persistent arterial hypotension [target mean arterial pressure (MAP) 50 mmHg]. Arriving at the hemodynamic laboratory an intracavital pacemaker electrode was led to the right ventricle through the right femoral vein due to complete atrioventricular block. After that, she still had no spontaneous circulation, thus coronary angiography was performed with ongoing mechanical chest compressions. It revealed an 80% stenosis in the middle segment of the left anterior descending artery (LAD) and a distal occlusion of the dominant right coronary artery. Coronary angioplasty was carried out on both arteries with stent implantations (Figures 1a,b).

After the procedure, ROSC was achieved and mechanical chest compressions could be terminated. The total duration of mechanical chest compressions was 2 h and 35 min. The arterial blood gas analysis (BGA) performed after the PCI (at 13:20 pH: 7.19, pCO_2_: 34.4 mmHg, pO_2_: 103.8 mmHg, SO_2_: 98%, HCO_3_: 13 mmol/l, BE: −14.2 mmol/l) compared to the one which was made at admission (at 11:41 pH: 6.82, pCO_2_: 35.4 mmHg, pO_2_: 190.7 mmHg, SO_2_: 99.9%, HCO_3_: 5.5 mmol/l, BE: −27.58 mmol/l) proves a clear improvement in the patients' metabolic condition. The patient was still at the hemodynamic laboratory when atrial fibrillation and systolic heart failure developed, thus intra-aortic balloon pump (IABP, Arrow International Inc., 2400 Bernville Rd, Reading, PA 19605-9607, USA) was inserted, and the patient was moved to the intensive care unit (ICU) with continuous circulatory support. Targeted temperature management (TTM) was initiated (33°C). In order to treat persistent arterial hypotension (MAP: 80 mmHg), infusion of norepinephrine (1 mg with a rate of 3 ml/h) and dobutamine (250 mg with a rate of 6 ml/h) were administered. Echocardiography revealed inferobasal akinesis of the left ventricle, nevertheless, ejection fraction (EF) was calculated to be 60%. Pericardial fluid was excluded. No fracture or pulmonary infiltrate was observed on the chest X-ray. After 3 days, the catecholamine support could be terminated and the IABP was removed. After finishing pacemaker treatment, a progressive heart failure appeared. Repeated echocardiography showed circumferential pericardial hematoma with a width of 20 mm, resulting in cardiac tamponade requiring urgent surgical repair (600 ml blood was drained, the hematoma was evacuated). The intervention led to a quick improvement in the patient's hemodynamic parameters. On hospital day 4, cranial computer tomography (CT) demonstrated cerebral edema and a hypodense area in the parasagittal lane within the cerebral hemisphere corresponding to a recent vascular lesion. No signs of midline shift were detected, but a small amount of blood could be observed along the tentorium. Owing to the risk of imminent herniation, the patient received dehydration with mannitol infusion (100 mg q.i.d. for 7 days). Despite the hemorrhagic cerebral lesions, platelet aggregation inhibitor as well as heparin therapy (100 mg of acetylsalicylic acid and 150 mg of clopidogrel/day, low molecular weight heparin 2 × 4000 NE) proceeded in consideration of the intracoronary stents. For the prevention of vasospasm, calcium channel blocker nimodipine along with abundant hydration was recommended by the neurologist; furthermore, the possibility of bilateral hemispheric dysfunction on account of the altered level of consciousness (coma) was raised. Afterwards, a slow improvement appeared in the patient's neurological condition (Table 1).

**Table 1:** Timeline of the case, condition of our patient.

	**Day 1 (arrival)**	**Day 4**	**Day 8**	**Day 16 (discharge)**
GCS	1-1-1	1-1-1	4-6-5	4-6-5
CPC score	4	4	3	1
Facial paresis	Not testable	Not testable	Mild right-sided central	Not detected
Paresis	Not testable	Tetraplegia	Proximal muscles of upper limbs: moderate paresis Lower limbs: mild paresis	Proximal muscles of upper limbs: mild paresis
Consciousness	Coma	Coma	Alert, disoriented in time	Alert, oriented in time and place
Left ventricular ejection fraction (%)	60	56	50	60
TAPSE (mm)	24	22	18	18

*GCS, Glasgow Coma Scale; TAPSE, tricuspid annular plane systolic excursion*.

On hospital day 7, the patient was successfully extubated and was able to follow simple commands. A repeated CT scan showed a hypodense lesion of 4 cm in the area of the trigonum. In the parieto-occipital region, a hypodense band was detected in the parasagittal plane within the territory of the posterior cerebral artery (PCA). It was considered to be an ischemic lesion within the territory of PCA. Signs indicative of cerebral aneurysm were not discovered. Compared to the previous scan, no significant change was detected. On the same day, right-sided homonymous hemianopia, mild right-sided central facial palsy, mild dysphonia, hypotonic limbs and moderate paresis in the proximal muscles of the upper extremities were observed. Although the patient was alert, spatial and temporal disorientation could be detected. In order to control her agitation, meprobamate was administered (200–200–400 mg). The dehydration was no longer continued. She subsequently received 6 g of piracetam daily and 30 mg nimodipine six times a day. Transthoracic echocardiography, performed on the eighth day of admission, revealed inferobasal left ventricular akinesis, while the remainder segments seemed hyperkinetic. Further echocardiographic examinations did not discover a significant reduction either in the EF or in the tricuspid annular plane systolic excursion (TAPSE) describing the right ventricular systolic capacity.

Because of temporary febrile state after the termination of TTM and elevated inflammatory markers (CRP: 152.83 mg/L, WBC: 13.53 G/L) antibiotic (1.2 g of intravenous amoxicillin + clavulanic acid t.i.d.) treatment was also provided from the second day of admission. After that, based on hemoculture results, amoxicillin was replaced with gentamycin, which was maintained until the normalization of the inflammatory parameters. Since anemia developed (hemoglobin level dropped from 100 to 86 g/L), the patient received a total of 5 units of B+ red blood cell transfusion within 6 days. Pericardial and intracranial bleedings were held responsible for the worsening anemia. Mobilization was implemented successfully and residual neurological symptoms were no longer detectable. Our evaluation was extended to the results of the Cerebral Performance Category scale (26).

Previous abnormalities found in her laboratory tests returned to normal levels, so the patient was discharged home in a good general state of health, after 16 days of inpatient care. She was recommended to give up smoking, attend follow-ups and take her medicines (100 mg of acetyl salicylic acid, 75 mg of clopidogrel, 5 mg of bisoprolol, 5 mg of perindopril b.i.d., 10 mg of rosuvastatin, 10 mg of amlodipine and 40 mg of pantoprazole) as prescribed.

Our patient's first follow-up examination occurred 1 year after being discharged, when she presented with a half-year history of atypical chest pain. During echocardiography, a I-II. degree tricuspid insufficiency was recognized, the left ventricular EF was 60% and the wall motion abnormality was no longer detectable. No atrial fibrillation or other arrhythmias were recognized. Considering her complaints and past medical history, exercise tolerance test was carried out, whereby the patient's resting ST depressions became more significant. Repeated coronary angiography visualized patent coronary stents and an 80% stenosis in the middle of the right coronary artery. Consequently, angioplasty was performed and a coronary stent (3.5 × 12 mm REBEL^TM^ stent, inflation pressure: 16 atm) was placed without complications.

## Discussion

In the setting of sudden cardiac arrest, the chance of survival is warranted by the early CPR, while the success of resuscitation is ensured by the quality and continuity of chest compressions (27–31). Previous studies verified that maintaining coronary perfusion pressure (CPP) during the resuscitation was an essential condition for the ROSC (28). Mechanical compression devices (LUCAS-2, LUCAS-3, AutoPulse) are capable of providing high-quality, uninterrupted and hands-free chest compressions, and ensure further necessary interventions. Liao et al. demonstrated that the coronary and cerebral perfusion pressure was significantly higher in the group resuscitated with the LUCAS mechanical device compared to the manual control group. Mean CPP in the case of the LUCAS-CPR was 20 mmHg and the cerebral perfusion pressure was 65 mmHg, as opposed to the manual group, where they measured 17 and 40 mmHg, respectively (32). In contrast, relevant studies, such as the CIRC (33), PARAMEDIC (2, 34), LINC trials (35) and the large clinical study carried out by Hallstrom et al. (36) did not find differences between the two methods in either the short-term outcome or the 30-day survival. The neurological condition of patients was also compared, but an obvious benefit regarding mechanical devices was not revealed. In another meta-analysis, results of 11,771 patients with regard to the ROSC were evaluated. Among 8 studies, only 3 (*n* = 300) found a benefit for the mechanical device [Dickinson, 1998: 14.3 vs. 0%; relative risk (RR): 4.13, 95% confidence interval (CI): 0.19–88.71; Lu, 2010: 55.3 vs. 37.8%; RR: 1.46; 95% CI: 1.02–2.08; Gao, 2016: 44.9 vs. 23.4%; RR: 1.92, 95% CI: 1.15–3.21]. Interestingly, four studies (*n* = 7,240) did not show significant differences between the groups. In contrast, in the CIRC trial (*n* = 4,231), the use of a mechanical device was associated with lower chances of the ROSC (RR: 0.88; 95% CI: 0.81–0.97). Rubertsson and Hallstrom examined the 24-h survival between manual and mechanical CPR groups and did not find any difference (*p* < 0.99 and *p* = 0.62, respectively). Further investigations on neurological outcome did not discover differences, while Hallstrom found less favorable results in the mechanical group (*p* = 0.006) (33, 35–38).

The injuries caused by mechanical compression devices have also been studied (37, 39). Smekal et al. analyzed the data of 222 patients, out of which 83 patients received manual compressions, while 139 patients were resuscitated with a compression device. In the manual group, there were 53 (64.6%) costal fractures and 45 (54.2%) sternal fractures, while in the mechanical group, 108 (78.8%) costal fractures and 81 (58.2%) sternal fractures were detected (*p* = 0.01 and *p* = 0.555). As for further injuries, a total of 59 cases of mediastinal and retrosternal hemorrhages were registered in the mechanical group, while in the manual group, injuries of this kind occurred in 27 cases. Ondruschka et al. performed an analysis of forensic autopsy reports of 614 patients who underwent unsuccessful resuscitation (manual group 501 vs. mechanical group 113 patients). No statistically significant difference was observed in the severity of injuries between the two groups (*p* = 0.09). Advanced age and prolonged resuscitation were associated with a higher incidence of costal and sternal injuries (*p* < 0.001). Hemothorax (*p* = 0.047), pneumothorax (*p* = 0.008), hemopericardium (*p* = 0.025), pulmonary (*p* = 0.008) and hepatic injuries (*p* = 0.001) were considerably more frequent in resuscitations with a mechanical device (40).

The results of studies are often incomplete in connection with injuries, which should also be taken into account, since different methods (e.g., autopsy, CT, ultrasound, X-ray) used in the evaluation of injuries highly influence their results. The patient's age and duration of resuscitation also have a substantial impact on the incidence of injuries. Furthermore, the application and deployment process of the device may confuse the providers, resulting in higher chances of injuries (33, 35–37).

At present, European Resuscitation Council (ERC) does not recommend the routine out-of-hospital use of mechanical chest compression devices; however, it highlights certain circumstances (e.g., hypothermic patients, during coronary intervention) which justify the use of such devices (4).

## Conclusion

Mechanical device can undoubtedly play a crucial role in the survival of patients by delivering high-quality chest compressions and maintaining adequate coronary and cerebral perfusion. Survival of this patient without a neurological deficit also proved that chest compressions performed by LUCAS, even over a long period of time, can be effective and may ensure adequate oxygenation. Long-term conclusions cannot be taken by one case, but in our opinion, usage of these devices create an opportunity to perform other interventions even during ongoing CPR, that can increase the survival rate. Further studies should clarify the exact role of mechanical compression devices in the primary emergency care.

## Data Availability Statement

The original contributions presented in the study are included in the article/supplementary material, further inquiries can be directed to the corresponding author/s.

## Ethics Statement

The studies involving human participants were reviewed and approved by Ethics Committee of the University of Debrecen (number of ethics approval: 16871-2016/EKU 0364/16). The patients/participants provided their written informed consent to participate in this study.

## Author Contributions

DU: collected, interpreted and analyzed patient data, and prepared the manuscript for publication. VS: analyzed and interpreted data and took part in preparing the manuscript. TÖ and IL: interpreted data and took part in revising the manuscript. BR: interpreted data and took part in preparing the manuscript. TS: collected cardiological data, prepared PCI pictures for publication and took part in revising the manuscript. ZC: interpreted cardiological data and took part in revising the manuscript. EB: collected and interpreted radiological data and took part in revising the manuscript. ZS: collected and interpreted data and prepared and reviewed the manuscript before publication. All authors contributed to the article and approved the submitted version.

## Conflict of Interest

The authors declare that the research was conducted in the absence of any commercial or financial relationships that could be construed as a potential conflict of interest.
